# Preoperative Coronary Anatomy Assessment with Echocardiography and Morbidity After Arterial Switch Operation of Transposition of the Great Arteries

**DOI:** 10.1007/s00246-018-1939-z

**Published:** 2018-07-12

**Authors:** Love Ahlström, Michal Odermarsky, Torsten Malm, Jens Johansson Ramgren, Petru Liuba

**Affiliations:** Department of Clinical Sciences Lund, Children Heart Center, Skane University Hospital, Lund University, Lund, 22185 Sweden

**Keywords:** Arterial switch operation, Coronary artery anatomy, Transposition of great arteries, Postoperative morbidity

## Abstract

In transposition of the great arteries (TGA), certain coronary patterns have been associated with major adverse events early after the arterial switch operation (ASO). We sought to determine the impact of preoperative echocardiographic (ECHO) diagnosis on the intra­ and postoperative morbidity. All patients with TGA born between June 2001 and June 2017 and who underwent ASO were reviewed. Data on presumed coronary anatomy (CA) preoperatively were obtained from the preoperative ECHO report. Intraoperative CA was categorized according to Yacoub classification. Major postoperative morbidity included at least one of the following: delayed sternal closure (DSC), prolonged (> 72 h) mechanical ventilation, reintubation, peritoneal dialysis (PD), ECMO, reoperation, and readmission within 30 days after surgery. 240 patients with median age of 5 days (range 1–614) and mean weight at surgery was 3.6 kg (1.8–8.4) were included. Preoperative ECHO assessment of CA was available in 228 patients. Intraoperatively, 181 patients (75%) were found to have type A, 25 patients had type B or C or intramural (B–C–IM; 10%), and 34 patients had type D or E (D–E; 14%). Patients with types B, C, and intramural coronary (B–C–IM) had increased risk for delayed sternum closure (9/25 vs. 20/181 in type A and 8/34 in type D–E; *p* = 0.04), peritoneal dialysis (4/25 vs. 8/181 and 1/34; *p* = 0.04), and ECMO (2/25 vs. 1/131 and 1/34; *p* = 0.02). Within the B–C–IM group, preoperative ECHO raised suspicion of type A in 13 patients (i.e., incorrect diagnosis, ID; 52%), whereas non-A CA was suspected in 12 patients (i.e., correct diagnosis, CD; 48%). With the exception of reoperation, which was seen only in the ID subgroup (4/12 vs. 0/10 in the CD subgroup; *p* = 0.04), the intraoperative (cardiopulmonary bypass time and cross-clamp time) and postoperative morbidity indices were comparable in both ID and CD subgroups (*p* > 0.1). Although there is a significant risk for early postoperative morbidity in TGA patients with single, interarterial, and intramural CA, there seems to be relatively limited influence of preoperative ECHO assessment of coronary anatomy on this morbidity burden.

## Introduction

Transposition of the great arteries (TGA) is a congenital heart defect that, in terms of both diagnosis and surgical management, has undergone important advances with significant improvement in mortality and morbidity. A major step in this regard has been the adoption of the arterial switch operation (ASO) instead of the previously used atrial switch [1]. ASO is an anatomical correction; the aorta and pulmonary trunk are switched and the coronary arteries are transferred to the neo-aorta.

To understand and improve outcome of TGA patients, several studies have investigated risk factors for postoperative mortality and morbidity. Thus, recognized factors for mortality and morbidity include institutional and surgeon’s inexperience, prematurity, lower weight at ASO, presence of ventricular septal defect, and left ventricle outflow obstruction including arch anomaly [2–9]. One additional risk factor involves the surgical translocation of the coronary arteries [1]. Certain types of coronary anatomy (CA) have been suggested to influence surgical indices such as bypass and aortic clamping time as well as early postoperative morbidity. Coronary ostium stenosis and even cardiac sudden death have been reported several years after surgery [10–14].

There are a few ways to categorize the CA in TGA. A commonly used classification is based on Yacoub`s studies where the anatomy is divided in 5 subgroups; type A to E [15]. The most common pattern, encountered in nearly two-thirds of the cases, is type A, meaning that the right and left coronary arteries arise from the middle of the right and left posterior aortic sinuses, and curve forwards to reach the right atrioventricular groove and anterior interventricular groove, respectively. The coronary patterns of intramural, type B (single coronary) and type C (two ostium close to each other associated with abnormal looping) have been linked with increased risk of postoperative mortality and morbidity [11, 16].

The preoperative diagnosis of TGA is typically based on transthoracic echocardiography (ECHO) and includes assessment of coronary anatomy [3, 17, 18]. Despite important advances in ultrasonic technology, ECHO identification of the origin of coronary arteries and their proximal course remains as a relatively difficult task in some cases. It requires strong expertise in ECHO and, relatively often, is also time-consuming. One study reported that preoperative ECHO had 81% accuracy of CA diagnosis, compared to 86% when angiography was used [18]. Another study showed an increase of sensitivity for type A diagnosis from 68 to 86%, and specificity from 92 to 94% when a two-reviewer method was used. The accurate ECHO diagnosis of all CA types rose from 68 to 75% [19].

Whether an accurate CA diagnosis improves ASO outcome has, to the best of our knowledge, not been investigated. We therefore sought to determine the impact of preoperative ECHO diagnosis on the intra­ and postoperative morbidity.

## Methods

### Study Population

Clinical records of all patients with TGA, with or without ventricular septal defect (VSD), born between June 2001 and June 2017 and treated at Skane University Hospital were reviewed. Medical data until discharge, including readmission within 30 days, were retrieved from the electronic patient chart system or from the paper-based medical records available in the hospital archive. Inclusion criteria were patients with TGA who underwent primary ASO at Skane University Hospital, and born during the above time interval. Exclusion criteria were double outlet right ventricle, outflow tract obstruction, chromosome abnormalities, and associated non-cardiac diseases.

### Definitions

Hemodynamically significant VSD was defined as either surgically closed VSD or by need of banding of the pulmonary artery at the time of ASO. Restrictive atrial septal defect was defined by need for balloon atrioseptostomy prior to ASO. Hospital mortality was defined as death within 30 days of the ASO. Major postoperative morbidity (MPM) was defined as the presence of 1 or more of the following: MV > 72 h, delayed sternum closure, reoperation, need of non-invasive ventilation post extubation, use of extracorporeal membrane oxygenation (ECMO) after ASO, peritoneal dialysis, and readmission within 30 days after discharge. Reoperation was defined as any surgical reintervention within the first month after ASO.

### Pre- and Postoperative Management and Surgical Technique

One hundred seventy-seven of 240 patients were diagnosed at hospitals other than Lund and seven patients were transferred from the Reykjavik University Hospital, Iceland. Median age at admission to Lund was 1 day. Fetal diagnosis of TGA was available in 59 cases. Prostaglandin infusion of 10–100 ng/kg/min was started either immediately after birth, when fetal diagnosis was available, or after postpartum diagnosis. ECHO of CA was attempted in all patients, as part of routine preoperative assessment. If unclear CA, ECHO was repeated typically by a senior cardiologist. All patients were admitted to either the pediatric or the neonatal ICU.

ASO was performed through median sternotomy and under cardiopulmonary bypass with hypothermia in all patients. In two cases, coronary arteries could not be transferred to the neo-aorta due to complex coronary anatomy. Eventual additional cardiac or extracardiac defects were repaired at the same stage. Three patients underwent a pulmonary banding during ASO due to a complex VSD and with a significant shunt that could not be surgically closed. Assessment of surgical result was performed by the pediatric cardiologist using transoesophageal or epicardial ECHO. Standard care during the first days after surgery included mechanical ventilation (MV), inotropic, diuretic, and analgesic support. New ECHO examination was routinely performed prior to extubation, and, if stable hemodynamics and spontaneous breathing were attained, patients were transferred to the pediatric cardiac ward for further care.

### Surgical Technique for Coronary Transfer

Aorta was transected 0.5–1 cm above the commissures and the coronary ostia were inspected and investigated for any abnormal anatomy. The coronary ostia were excised with a large D-shaped cuff. The pulmonary artery was transected and medial hinged trapdoor flaps were cut at the site of previously placed marking stitches. If the coronary ostia were placed eccentrically or were intramural, the valve commissure was excised and later resuspended to the pericardial patch that was implanted to cover the defects in the aortic sinuses. The coronary artery cuffs were transferred and re-implanted into the incisions producing the trapdoor flaps without creating any kinking, rotation, or inappropriate stretching. Techniques used to deal with non-A CA are described elsewhere [20].

### Data Collection

Data on presumed CA preoperatively were obtained from the surgeons preoperative evaluation and eventually compared with the pediatric cardiologist`s ECHO report. Intraoperative CA was categorized according to Yacoub classification in types A–E [15, 21]. Demographic, pre-, intra-, and postoperative data were collected including readmission within 30 days.

The preoperative ECHO diagnosis was assessed by the ability to distinguish type A from non-A CA. Secondly, preoperative ECHO was evaluated by the accuracy to predict the intraoperative CA, which was regarded as reference standard.

### Statistical Analysis

Categorical data are presented as counts and percentages, whereas numerical data are given as mean ± SD or, in case of variables with non-Gaussian distribution, as median and range. Either non-parametric test (Kruskal Wallis and Mann–Whitney) or ANOVA were used to compare numerical variables, whereas Chi-square test with Yates correction for continuity was used for categorical dichotomous variables. A *p* value of less than 0.05 was considered statistically significant. SPSS Statistics version 22.0 for Mac (IBM Corporation, Armonk, NY) and StatView 5.0 were used for data analysis.

## Results

### Demographic Characteristics

Two hundred forty-five patients matched the inclusion criteria. Of these, two patients had missing clinical records and were therefore excluded. Two patients were excluded due to total anomalous pulmonary venous drainage and Goldenhar syndrome (encompassing multiple extracardiac malformations), respectively. One premature baby born week 34 with birth weight 2 220 g and CA type A, developed severe myocardial failure shortly after ASO and died several days later on ECMO due to severe cerebral bleeding. This patient was also excluded from further analysis. Consequently 240 patients (girls *n* = 61) were reviewed. Median age at ASO was 5 days (range 1–614) and mean weight at surgery was 3.6 kg (1.8–8.4). Preoperative characteristics are summarized in Table 1.

**Table 1:** Preoperative characteristics of patient population

	Type A *n* = 181	Type B–C–IM *n* = 25	Type D–E *n* = 34	Total *n* = 240
Gender male	133 (74%)	17 (68%)	29 (85%)	179 (75%)
Gestational age (weeks)	39 (31–42)	40 (29–41)	39 (32–42)	39 (29–42)
Prematurity (< 37 weeks)	8 (4%)	2 (8%)	3 (9%)	13 (5%)
Intrauterine suspicion of TGA	45 (25%)	6 (24%)	8 (24%)	59 (25%)
Birth weight (kg)	3.5 ± 0.6	3.5 ± 0.6	3.5 ± 0.8	3.4 ± 0.6
Preoperative prostaglandin infusion use	166 (92%)	23 (92%)	32 (94%)	221 (92%)
Preoperative mechanical and assisted ventilation use	90 (50%)	12 (48%)	14 (40%)	116 (48%)
Balloon atrioseptostomy	100 (55%)	9 (36%)	20 (59%)	129 (54%)

Fourteen (5.8%) patients had extracardiac defects; in 13 patients (two with interrupted aortic arch, and 11 with aortic coarctation ± hypoplastic aortic arch), correction was achieved during ASO. In one patient, the coarctation was repaired at a later stage as it was initially judged as non-significant. Eighty-three (34.6%) had a VSD requiring repair. The intraoperative characteristics are summarized in Table 2.

**Table 2:** Intraoperative characteristics of the patients

	Type A *n* = 181	Type B–C–IM *n* = 25	Type D–E *n* = 34	Total *n* = 240
Age at surgery (days)	5 (1–614)	6 (1–66)	6.5 (1–123)	5 (1–614)
Weight at surgery (kg)	3.5 (1.8–8.4)	3.5 (2.3–5.3)	3.5 (1.9–5.3)	3.5 (1.8–8.4)
VSD requiring surgery	64 (35%)	11 (44%)	8 (24%)	83 (35%)
Corrected extracardiac defects	13 (7%)	1 (4%)	0	14 (6%)

### Preoperative Coronary Echocardiography and Intraoperative Coronary Anatomy

Preoperative ECHO data of CA were available in 228 patients; the remaining 12 patients had missing or unclear preoperative CA assessment. Type A was suspected in 196 patients and non-A CA in 32 patients.

Intraoperative findings identified 181 patients (75.4%) with type A, 25 patients (10.4%) type B or C or intramural (IM), and 34 patients (14.2%) with type D or E. Among the 196 patients with preoperative ECHO suspicion of type A, 33 patients (17%) had non-A CA. Among the 32 patients with suspect non-A CA, 8 patients (25%) were found to have type A. For type A, the preoperative ECHO had a sensitivity of 95.3% and a specificity of 42%.

Preoperative ECHO assessed CA was incorrect in 40 out of 228 cases. When comparing first and second half of the study period, there was no significant difference regarding the accuracy of diagnosing CA with preoperative ECHO (97 accurate preoperative ECHO of 121 vs. 91 of 107, *p* = 0.3).

### Coronary Anatomy and Postoperative Complications

There were in total 25 reoperations: four evacuation of pericardial fluid, seven pleural drainages, seven resternotomies due to postoperative bleeding, two pacemaker insertions, two ECMO, one cardiac arrest occurring shortly after delayed sternum closure, which required resternotomy and heart compressions, one operation due to deep infection in mediastinum, and one operation due to infection after preoperative ECMO.

Patients with types B, C, and intramural coronary (B–C–IM) had increased risk for delayed sternum closure (9/25 vs. 20/181 in type A and 8/34 in type D–E; *p* = 0.04), peritoneal dialysis (4/25 vs. 8/181 and 1/34; *p* = 0.04), and ECMO (2/25 vs. 1/131 and 1/34; *p* = 0.02). The remaining postoperative indices, including non-invasive ventilation, reoperation, and MPM-score, showed no significant difference (*p* ≥ 0.1). Similarly, there was no difference in intraoperative variables including cardiopulmonary bypass and cross-clamp time. Postoperative data are summarized in Table 3.

**Table 3:** Intra- and postoperative outcome in relation to CA subgroup

	Type A *n* = 181	Type B–C–IM *n* = 25	Type D–E *n* = 34	Total *n* = 240	*p* value (lowest between groups)
Cardiopulmonary bypass time (min)	184 (119–458)	171 (137–381)	173 (120–381)	181 (119–458)	0.9
Cross-clamp time, minutes	95 (63–269)	87 (46–154)	89 (59–186)	94 (46–269)	0.3
Mechanical ventilation (h)	40 (7.5–303)	45 (19–358)	44 (17–348)	42 (7.5–358)	0.2
Total ICU stay (days)	3 (1–14)	3 (2–31)	3 (2–21)	3 (1–31)	0.3
Postoperative hospital stay (days)	10 ± 3	10 ± 5	11 ± 6	10 ± 4	0.4
Delayed sternal closure^a^	20 (20%)	9 (36%)	8 (23%)	37 (15%)	0.001^#^
Peritoneal dialysis^a^	8 (4%)	4 (16%)	1 (3%)	13 (5%)	0.04^#^
Prolonged mechanical ventilation, > 72 h^a^	21 (11%)	7 (28%)	5 (15%)	33 (14%)	0.08
ECMO^a^	1 (1%)	2 (8%)	1 (3%)	4 (2%)	0.02^#^
Non-invasive ventilation post extubation^a^	30 (17%)	1 (4%)	5 (15%)	36 (15%)	0.3
Readmission within 30 days^a^	2 (1%)	0	1 (3%)	3 (1%)	0.6
Reoperations^a^	18 (10%)	4 (16%)	3 (9%)	25 (10%)	0.7
Major postoperative morbidity	56 (31%)	10 (40%)	11 (44%)	77 (32%)	0.7

^a^Indicates variables included in the major postoperative morbidity

^#^Indicates statistically significant differences between groups

### Impact of Preoperative Coronary Anatomy Diagnosis on ASO outcome

Within the B–C–IM group, preoperative ECHO raised suspicion of type A in 13 patients (i.e., incorrect diagnosis, ID; 52%), whereas non-A CA was suspected in 12 patients (i.e., correct diagnosis, CD; 45%). When comparing these two subgroups for intraoperative variables (cardiopulmonary bypass time and cross-clamp time), no difference was found (*p* = 0.5 for both). Regarding postoperative morbidity indices, reoperation was needed in 4/12 in the ID group versus 0/10 in the CD group (*p* = 0.04). The preoperative and postoperative characteristics are summarized in Tables 4 and 5.

**Table 4:** The demographics of incorrect and correct ECHO diagnosis

	Incorrect diagnosis *n* = 13	Correct diagnosis *n* = 12
Gender male	5 (38%)	3 (25%)
Gestational age (weeks)	39 (34–41)	39 (29–41)
Prematurity (< 37 weeks)	1 (8%)	1 (10%)
Intrauterine suspicion of TGA	4 (31%)	4 (25%)
Birth weight (kg)	3.5 (1.9-5,5)	3.4 (2,3–4,7)
Preoperative prostaglandin infusion use	13 (100%)	10 (83%)
Preoperative mechanical and assisted ventilation use	5 (38%)	7 (58%)
Balloon atrioseptostomy	5 (38%)	4 (25%)
Surgery age (days)	5 (3–13)	7 (1–66)
Corrected VSD	2 (15%)	9 (75%)
Corrected extracardiac defects	1 (8%)	0

**Table 5:** CA type B, C, and intramural: the outcome regarding incorrect or correct preoperative ECHO

	Incorrect diagnosis *n* = 13	Correct diagnosis *n* = 12	*p* value
Cardiopulmonary bypass time (min)	192 ± 73	212 ± 66	0.5
Cross-clamp time (min)	89 ± 26	95 ± 16	0.5
Mechanical ventilation (h)	32 (19–358)	48 (20–116)	0.7
Total ICU stay (days)	6 ± 8	3 ± 1	0.3
Major postoperative morbidity	5 (38%)	5/ (42%)	0.9
Peritoneal dialysis^a^	3 (23%)	1 (8%)	0.3
Reoperations^a^	4 (31%)	0	0.04 ^#^
Readmissions^a^	0	0	–
Delayed sternal closure^a^	4 (31%)	5 (42%)	0.6
Postoperative non-invasive ventilation^a^	1 (8%)	0	0.3
ECMO^a^	2 (15%)	0	0.2
Mechanical ventilation > 72 h^a^	4 (31%)	3 (25%)	0.7

^a^Indicates variables included in the major postoperative morbidity

^#^Indicates statistically significant differences between groups

In the ID subgroup, 1 patient had aortic coarctation, which was treated with end-to-end anastomosis, and also large apical VSD, which was palliated with pulmonary banding. Remaining demographic variables were evenly distributed between the groups.

## Discussion

This retrospective survey conducted on a large cohort of TGA patients with ASO confirms the previously reported association of certain non-A types of coronary anatomy, i.e., single (type B), interarterial (type C), and intramural (IM) CA, with increased early postoperative morbidity. Even though the accuracy of preoperative ECHO diagnosis for non-A CA was fairly modest, ECHO appeared to have only minor effect on postoperative outcome. Thus, with the exception of the risk for reoperations, which was mildly increased, no other index of early postoperative morbidity was associated with failure to correctly diagnose non-A CA by ECHO.

Given the complexity of ASO as surgical method, the preoperative assessment includes thorough investigation of CA. With the advance of ultrasound technique, echocardiography is increasingly used in the preoperative diagnosis of CA in neonates with TGA. Coronary ECHO is though time-consuming and requires in addition to modern ultrasound equipment a high level of training and expertise. Thus, we sought to assess the accuracy and importance of preoperative coronary ECHO on several indices of intra- and postoperative morbidity. Since only one patient died during the studied time interval, no conclusions can be drawn in with regard to the impact of coronary ECHO on mortality.

As some types of CA have been linked to both immediate and late complications after ASO, the purpose of preoperative coronary ECHO is to increase the awareness of surgical team with respect to eventual high-risk CA. Therefore, we divided the cohort based on preoperative CA echo in type A and non-A. ECHO diagnosis of CA has highest sensitivity for type A (18). Assessment of CA is far more difficult in non-A types, especially in patients with intramural and single coronaries [19].

Previous TGA studies of CA assessment with ECHO have reported a sensitivity for CA diagnosis of 60–90% [18, 19, 22, 23] which is comparable to our study. Gremmels et al. introduced an independent double-observer approach that increased the accuracy rate from 68 to 86% [19]. McMahon et al. found that preoperative ECHO diagnosis of CA is comparable with angiography [18]. Pasquali et al. speculated that the preoperative imaging, along with operator experience, has likely contributed to decreased mortality in patients with intramural CA [11]. Chen et al. emphasized the importance of preoperative diagnosis of IM type, although none of the seven patients with IM was diagnosed preoperatively [24]. Baslaim studied if preoperative delineation of CA is a prerequisite for ASO. Of 66 patients with TGA or double outlet of right ventricle, 40% had a correct preoperative ECHO while remaining had wrong or missing preoperative assessment [25]. The authors concluded that trapdoor flap and circular buttonhole transfer techniques in coronary transfer could compensate for limited preoperative ECHO of CA. From our point of view, this study does not give an answer if ECHO influence outcome. On the basis of these surveys it is tempting to believe that correct ECHO would lower morbidity burden in AD group, which our result nearly failed to do. A speculation of the surgeon’s intraoperative judgment of CA and adequate transfer technique is fundamental for good outcome in the aspect of CA.

The present findings indicate increased early postoperative morbidity in patients with types B–C–IM but similar intraoperative indices compared with patients with type A. Most of the previous reports of CA have mainly focused on postoperative mortality, however, such analysis was not possible in our study due to only one death. Previous meta-analysis of CA and outcome showed that intramural coronaries have an approximately six-fold higher mortality (OR 6.5), single coronary with a loop has threefold increased risk (OR 2.9), whereas CA with looping coronaries around the great vessels from different ostium were not linked to increased hospital death [11]. This observation was confirmed by later studies; Metton et al. reported increased mortality for intramural CA that was independent of time-trends adjustment [26]. Legendre et al. showed that CA types B and C were significantly correlated to coronary events [16]. However, other studies have failed to confirm an association between abnormal CA and increased mortality [7, 14, 24].

From a morbidity perspective, intramural coronaries have been correlated with higher incidence of intra- and postoperative coronary problems requiring interventions [14] and inverted and single coronary have been associated with delayed sternal closure, longer MV, and CPB time [6]. Why type B and C (both have a coronary course between the aorta and the pulmonary artery) and intramural CA (part of the coronary inside the aortic wall) are associated with morbidity is not fully understood. In our surgical series, neither demographics nor intraoperative variables varied between CA groups. Pasquali et al. speculate that the vulnerable factor for single coronary is the sole source of blood flow to the whole cardium and consequently, if any events of kinking, stretching, or thrombosis will have a profound effect [11]. Several researchers have accounted surgical difficulties for the increased risk of morbidity related to intramural coronaries [14, 24, 26].

### Limitations

As with all retrospective studies, there are inherent limitations that caution carefulness in interpretation. Although the main cohort has a relevant size, the subgroup B–C–IM is small and could neutralize any significance even though we failed to find any. Clearly, further large prospective studies are warranted.

## Conclusions

Although there is a significant risk for early postoperative morbidity in TGA patients with single, interarterial, and intramural CA, there seems to be relatively little influence of preoperative ECHO assessment of coronary anatomy on this morbidity burden. Refining of ECHO technique for better delineation of especially non-A CA is nevertheless important. Further prospective studies are warranted to better investigate the association of preoperative ECHO diagnosis with postoperative morbidity and mortality. Until future studies have elucidated the impact of ECHO, surgeons need to be aware of the relative inaccuracy of ECHO in CA assessment.

All procedures performed in studies involving human participants were in accordance with the ethical standards of the institutional and/or national research committee and with the 1964 Helsinki Declaration and its later amendments or comparable ethical standards. For this type of study, formal consent is not required.

## Abbreviations

ASO: Arterial switch operation
CA: Coronary anatomy
CD: Correct diagnosis
DSC: Delayed sternal closure
ECHO: Echocardiography
ECMO: Extracorporeal membrane oxygenation
ICU: Intensive care unit
ID: incorrect diagnosis
IM: Intramural
MPM: Major postoperative morbidity
MV: Mechanical ventilation
PD: Peritoneal dialysis
TGA: Transposition of the great arteries
VSD: Ventricular septal defect
